# Identification of Candidate Causal Polymorphisms in *GGT1* and *SLC5A1* Associated with Fat Area Ratio on BTA17 in Japanese Black Cattle

**DOI:** 10.3390/genes17040363

**Published:** 2026-03-24

**Authors:** Shinji Sasazaki, Hikari Ito, Ryoto Adachi, Eiji Iwamoto, Emi Yoshida, Fuki Kawaguchi, Kenji Oyama, Hideyuki Mannen

**Affiliations:** 1 Laboratory of Animal Breeding and Genetics, Graduate School of Agricultural Science, Kobe University, Kobe 657-8501, Japan; mokapi.ih@gmail.com (H.I.); a.ryoto.kobe@outlook.com (R.A.); kawaguchi@koala.kobe-u.ac.jp (F.K.); mannen@kobe-u.ac.jp (H.M.); 2 Hyogo Prefectural Technology Center for Agriculture, Forestry and Fisheries, Kasai 679-0198, Japan; eiji_iwamoto@pref.hyogo.lg.jp (E.I.); emi_yoshida@pref.hyogo.lg.jp (E.Y.); 3 Food Resources Education & Research Center, Kobe University, Kasai 675-2103, Japan; oyama@kobe-u.ac.jp

**Keywords:** GWAS, beef marbling, Japanese Black cattle, meat quality

## Abstract

**Background/Objectives**: Intramuscular fat deposition is a key determinant of beef quality in Japanese Black cattle, and the fat area ratio of the rib eye (FAR) is highly correlated with Beef Marbling Standard scores. **Methods**: To identify genetic variants underlying variation in the FAR, we conducted a genome-wide association study (GWAS) followed by whole-genome sequence–based fine mapping in a Hyogo Japanese Black population (*n* = 432). Animals were genotyped using the Illumina BovineSNP50v3 BeadChip, and association analysis was performed using residuals derived from a linear mixed model accounting for fixed and random effects. **Results**: A significant association signal was detected on BTA17 (λ = 1.09), with the top single nucleotide polymorphism (SNP) located at 17:72,329,662 (*p* = 3.60 × 10^−6^). To refine the candidate region, we analyzed whole-genome resequencing data from 42 Hyogo Japanese Black cattle and identified a distinct linkage disequilibrium (LD) block spanning 71–74 Mbp on BTA17. Among 4292 variants within genes showing LD (*r*^2^ ≥ 0.1) with the top SNP, 96 variants with strong LD and predicted functional effects were selected for validation. Genotyping in the Hyogo population revealed that a missense variant in *gamma-glutamyltransferase 1 (GGT1*) (c.589G>A, p.Asp197Asn) showed the strongest association with FAR (*p* = 3.89 × 10^−6^). A 5′UTR variant in *GGT1* (c. −256G>T) and a missense variant in *solute carrier family 5 member 1 (SLC5A1*) (c.32C>T, p.Thr11Met) also exhibited significant associations and strong LD with the top SNP (*r*^2^ > 0.7). GGT1 is involved in glutathione metabolism, whereas *SLC5A1* encodes a sodium–glucose cotransporter implicated in nutrient sensing and metabolic regulation. **Conclusions**: Although functional validation is required, these variants represent strong positional and biological candidates underlying the BTA17 quantitative trait loci (QTL). The identified polymorphisms may provide useful molecular markers for optimizing genetic improvement of marbling-related traits within the Hyogo Japanese Black population.

## 1. Introduction

Intramuscular fat, commonly referred to as beef marbling, is a critical determinant of beef quality, as it significantly influences the melt-in-the-mouth texture and palatability of the meat. Japanese Black cattle, the most important Wagyu breed in Japan, are well known for their exceptional genetic potential for intramuscular fat deposition [1]. This breed has been intensively selected for meat quality traits for several decades, resulting in distinctive characteristics such as high marbling ability, relatively high heritability of carcass traits, and unique genetic structure compared with other beef breeds [2,3]. Furthermore, Japanese Black cattle have often been maintained in relatively closed breeding populations, leading to distinctive genetic backgrounds that make this breed particularly valuable for genetic studies of meat quality traits [4,5]. Consequently, numerous genetic studies have been conducted in Japanese Black cattle to identify loci affecting carcass and meat quality traits, particularly those related to marbling. However, in recent years, shifts in consumer health consciousness and the diversification of preferences have increased the demand for more precise genetic control of fat deposition—not merely increasing marbling, but adjusting it to moderate levels. In Japan, beef marbling is evaluated using the Beef Marbling Standard (BMS), a scoring system traditionally conducted by trained graders via visual inspection. Because BMS integrates multiple components, including overall fat content and fat particle fineness, elucidating the specific factors influencing these components is essential for precise genetic improvement. This study focuses on the fat area ratio (FAR), an image-derived quantitative measure representing the proportion of intramuscular fat within the ribeye (longissimus muscle) area, which exhibits a strong positive correlation (*r* = 0.81–0.99) with BMS scores [6,7].

Beef marbling is known to be under substantial genetic control. Heritability estimates for marbling vary among breeds, with reported values of 0.31 in Nellore [8], and 0.48 and 0.37 in Angus and Brahman, respectively [9]. Notably, Japanese Black cattle exhibit an even higher heritability of 0.72, despite decades of intensive selection [10]. Similarly, the FAR has demonstrated a high heritability of 0.59 [11], underscoring the efficacy of genetic strategies for improving this trait. Extensive research has aimed to identify the quantitative trait loci (QTLs) and specific genes associated with beef marbling. For instance, a polymorphism in the 5′UTR of the *endothelial differentiation, sphingolipid G-protein-coupled receptor 1* (*EDG1*) gene has been significantly associated with marbling, potentially influencing gene expression levels [12]. Additionally, a single nucleotide polymorphism (SNP) in the *promoter region of the ribosomal protein L27a* (*RPL27A*) gene was found to correlate with breeding values for marbling [13]. Multiple QTLs affecting marbling have been reported across several chromosomes, including BTA 6, 7, 9, 10, 20, and 21 [14,15,16,17]. Despite these findings, the primary causative genes and functional polymorphisms remain largely elusive, posing a significant challenge for the precise modulation of marbling. Recent studies have suggested that genes involved in oxidative stress regulation and glucose metabolism may also influence adipogenesis and fat deposition. For example, gamma-glutamyltransferase 1 (GGT1) is involved in glutathione metabolism and oxidative stress regulation, processes that may influence lipid metabolism and insulin sensitivity [18,19]. Similarly, solute carrier family 5 member 1 (SLC5A1) encodes a sodium–glucose cotransporter involved in nutrient absorption and metabolic regulation, which may affect adipocyte differentiation and energy metabolism [20,21]. However, the potential involvement of these genes in marbling-related traits in Japanese Black cattle remains largely unexplored.

Recent advancements in genomic technologies have enabled the exploration of causative polymorphisms using comprehensive sequence data for various quantitative traits in cattle [22]. Using whole-genome resequencing data combined with functional annotation and linkage disequilibrium (LD) analysis, we previously identified candidate genes and polymorphisms underlying QTLs for fatty acid composition on BTA 6 [23]. Furthermore, a similar approach was applied to FAR, identifying candidate polymorphisms within a QTL on BTA 7 [24]. Validation of these candidates in a Miyazaki Prefecture population led to the identification of the *intercellular adhesion molecule 1* (*ICAM1*) gene, which has been implicated in adipocyte differentiation, as a strong candidate for marbling, with two missense mutations (c.470C>G and c.994G>A) identified as the likely functional variants [25].

These methodologies demonstrate that leveraging comprehensive variant data is highly effective for the efficient identification of causative polymorphisms. Building on these previous findings, the present study performs a genome-wide association study (GWAS) followed by an exhaustive search for candidate polymorphisms associated with FAR in a Japanese Black cattle population from Hyogo Prefecture, a major Wagyu-producing region with distinct breeding lines. Given that the heritability of FAR in this population is estimated at 0.592 [7], there is significant potential for further genetic improvement. This study aims to identify novel causative polymorphisms underlying FAR, thereby providing robust molecular markers for optimized genetic selection.

## 2. Materials and Methods

### 2.1. Animals

We used 432 Japanese Black cattle from Hyogo Prefecture (343 steers and 89 heifers). They were randomly selected from a total of 1836 animals, which were slaughtered at 31.9 months of age on average during 2010–2012 [7]. The population consisted of progeny from seven sires, each with at least 10 offspring. Genomic DNA was extracted from 50 mg samples of longissimus cervicis muscle using the standard phenol–chloroform method. All animals were raised under standard commercial management conditions in Hyogo Prefecture prior to slaughter.

The target trait, the FAR, was measured by image analysis. To analyze FAR, high-quality digital images of the carcass cross-section were taken between the sixth and seventh ribs by photographing equipment developed by the previous study [11]. FAR was calculated as the percentage of pixels classified as fat within the longissimus muscle area divided by the total number of pixels within the same area. The rib-eye image was binarized as lean and fat using the image analysis program. The binarized image was subjected to 10 iterations of thinning, followed by removal of hairline artifacts. Ultimately, FAR was defined as the proportion (%) of fat area relative to the total area of the longissimus muscle.

### 2.2. GWAS

All 432 animals were genotyped using the Illumina BovineSNP50v3 BeadChip (San Diego, CA, USA), which includes 53,217 SNP markers. Quality control procedures were applied to exclude SNPs with a minor allele frequency < 0.01, call rate < 0.95, or deviation from Hardy–Weinberg equilibrium (*p* < 0.001). After filtering, a total of 29,568 SNPs located on 29 autosomal chromosomes remained for association analysis.

We performed variance component estimation using 1836 animals and their 6825 ancestors. The phenotypes were pre-adjusted following the method described in a previous study [7], applying a linear mixed model to FAR as follows:

**y** = **Xb** + **Z**_1_**u**_1_ + **Z**_2_**u**_2_ + **e**

where **y** is the vector of observations (FAR); **b** is a vector of fixed effects, including the overall mean, slaughter year, slaughter month, sex, a linear covariate for inbreeding coefficient, and linear and quadratic covariates for age at slaughter; **u**_1_ and **u**_2_ are vectors of random farm (62 levels) and additive genetic (animal) effects, respectively; **e** is a vector of random residuals; and **X**, **Z**_1_, and **Z**_2_ are known incidence matrices. Restricted maximum likelihood (REML) estimation and best linear unbiased prediction were used to estimate variance components and all random effects, respectively. After estimating these effects, we calculated the residuals (e-values) as follows:

$$\hat{e}=y-(X\hat{b}+Z_{1}{\hat{u}}_{1}+Z_{2}{\hat{u}}_{2})$$

A Wald test was performed using the e-values of 432 genotyped animals, and asymptotic *p*-values were obtained with PLINK software v2.0 [26]. *p*-values from the analysis were adjusted using the genomic control method. The Bonferroni correction was applied to account for multiple hypothesis testing to identify genome-wide significant (0.05/29,568) and suggestive (1/29,568) SNPs.

### 2.3. Detection of Polymorphisms Within the Candidate Region

All polymorphisms within the candidate region (chr17: 71–74 Mbp) were identified by comparing the bovine reference genome sequence (UMD3.1) with whole-genome sequencing (WGS) data from 42 Japanese Black cattle of Hyogo which were the progeny of 30 sires and slaughtered in 2010–2012 (*n* = 27) or 2016–2019 (*n* = 15). 11 of them were also included in the population used in the GWAS analysis (*n* = 432). The whole-genome sequencing in these 42 animals was conducted in a previous study [23].

### 2.4. Linkage Disequilibrium Analysis

Using whole-genome sequencing data, we focused on biallelic polymorphisms located from 68 Mb on chromosome 17 to the distal telomere. Polymorphisms for which genotypes were not successfully determined in at least one of the 42 individuals subjected to whole-genome sequencing were excluded from subsequent analyses. To verify LD structure around the top SNP, LD coefficients (*r*^2^) between the top SNP and these polymorphisms were calculated using HAPLOVIEW 4.0 using the whole-genome sequencing data. The top SNP passed standard genotype quality control criteria, including a high call rate (>99%), no significant deviation from Hardy–Weinberg equilibrium, and clear genotype clustering upon visual inspection.

### 2.5. Verifying the Effects of Candidate Polymorphisms on FAR

Among the variants showing LD with the top SNP (*r*^2^ ≥ 0.1), we extracted those located within genes in the candidate region. This relatively permissive threshold (*r*^2^ ≥ 0.1) was adopted to capture potential causal variants within the extended LD structure observed in Japanese Black cattle, particularly in the Hyogo population, which has experienced strong artificial selection and a relatively small effective population size. These variants were then evaluated as potential causal polymorphisms based on their LD (*r*^2^) with the top SNP and their functional annotations (e.g., missense_variant, 5_prime_UTR_variant, splice_region_variant, and upstream_gene_variant). Specifically, variants exhibiting stronger LD with the top SNP and annotations more likely to affect gene function were considered stronger candidate causal variants. Based on these criteria, the top 96 variants were selected for association analysis with FAR in the Hyogo Japanese Black cattle population. However, when multiple variants located within the same gene showed identical *r*^2^ values with the top SNP, only several representative variants were selected for further analysis (Table S1).

The selected polymorphisms were genotyped in the Hyogo population (*n* = 432) using either the BiomarkTM HD system (Standard BioTools, South San Francisco, CA, USA) or the Kompetitive Allele Specific PCR (KASP) assay (LGC Genomics, Hoddesdon, UK). Primer and probe sequences used for genotyping are listed in Table S1. Genotyping was performed according to each manufacturer’s protocol. Associations between candidate polymorphisms and FAR were evaluated using one-way analysis of variance (ANOVA), with genotype treated as a categorical factor. Adjusted FAR residuals obtained from the animal model were used as the response variable. For variants showing significant overall genotype effects, genotype-specific least-squares means were compared using Tukey–Kramer’s honestly significant difference (HSD) test.

### 2.6. Gene Function Research

Within the candidate region (chr17: 71–74 Mbp), we investigated the functional relevance to beef marbling of all 60 genes harboring variants in linkage disequilibrium with the top SNP (*r*^2^ ≥ 0.1). Functional information for each gene was comprehensively retrieved from the Gene and PubMed databases of the National Center for Biotechnology Information (NCBI; https://www.ncbi.nlm.nih.gov/) as well as from GeneCards (https://www.genecards.org/). Based on the collected functional annotations and published evidence, genes were considered functionally relevant if published evidence supported their involvement in adipogenesis, lipid metabolism, or adipocyte maturation.

## 3. Results and Discussion

**Figure 1:**
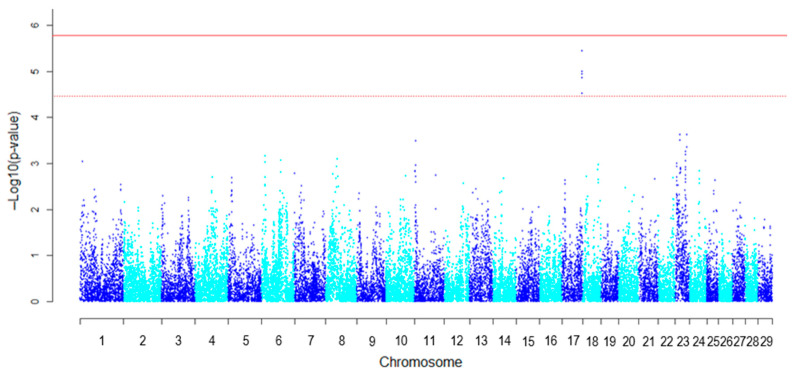
Genome-wide association plot of −log_10_ (*p*-value) for loci associated with the basis of fat area ratio to rib eye area (FAR) in the Hyogo population (*n* = 432). The *x*-axis represents chromosome numbers, and the *y*-axis shows −log_10_ (*p*-value). The solid line indicates the Bonferroni-corrected 5% genome-wide significance threshold (5.77), while the dashed line indicates the suggestive significance threshold (4.47).

The GWAS identified an association signal with five suggestive SNPs on BTA17, with a genomic inflation factor (λ) of 1.09 (Figure 1, Table 1). The SNP showing lowest *p*-value was located at 17:72,329,662 (*p* = 3.60 × 10^−6^) and was defined as the top SNP. Although numerous GWAS for beef marbling have been conducted in diverse cattle populations [27,28,29], to our knowledge, no previous study has reported a QTL for marbling-related traits within the chr17:71–74 Mb region. Differences in QTL positions among Japanese Black subpopulations have previously been documented [30]. Therefore, the QTL detected in this study may represent a locus specific to the Hyogo Japanese Black population. This population has been maintained under a closed breeding system for several decades, and previous studies have suggested that it exhibits distinctive genetic characteristics, including elevated inbreeding and genetic differentiation [4,5]. The detection of a unique QTL in the present study may therefore reflect its distinct genetic background.

**Figure 2:**
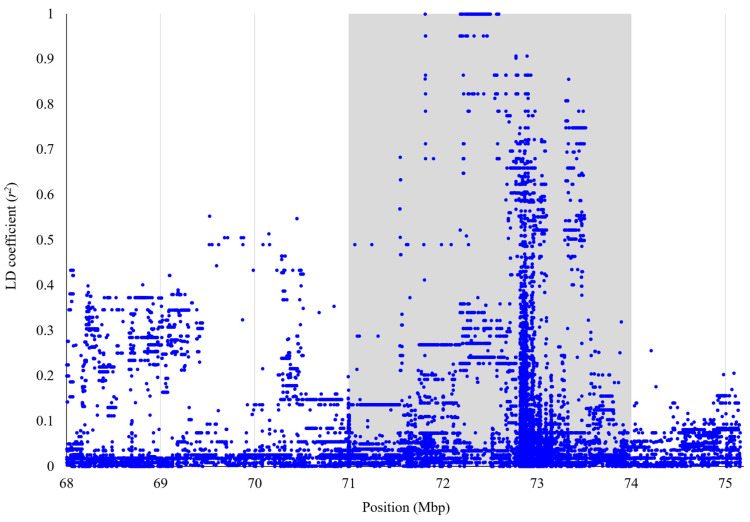
LD coefficients (*r*^2^) between top SNP and other polymorphisms around top SNP. *x*-axis represents locations of polymorphisms on BTA17 while *y*-axis represents *r*^2^-values. Gray shading indicates the candidate region (chr17:71–74 Mbp).

In typical Manhattan plots for quantitative traits in cattle, clusters of significant SNPs surrounding the most significant marker are commonly observed [27,28,29]. Such clustering is generally attributed to strong LD between the causal variant and nearby markers, and LD patterns are often used to estimate the boundaries of a QTL. In contrast, in the present study, only five SNPs (Table 1) showed strong statistical significance, whereas surrounding markers exhibited little evidence of association. To clarify the LD structure around the top SNP, we increased marker density using WGS data. Among polymorphisms successfully genotyped in all 42 sequenced Hyogo Japanese Black cattle, 33,979 biallelic variants were identified between 68 Mb and the distal telomere of BTA17. LD coefficients (*r*^2^) between these variants and the top SNP revealed a distinct LD block spanning 71–74 Mbp (Figure 2). Based on this pattern, the region chr17:71–74 Mbp was defined as the candidate region.

A total of 113 genes were located within this candidate region. Among variants with *r*^2^ ≥ 0.1 relative to the top SNP, 4292 variants were located within these genes (Table S2). Variant annotations provide information regarding predicted functional consequences. For example, missense variants, splice-region variants, and stop-gained variants may alter protein structure and function [31,32,33], whereas variants in untranslated regions or regulatory regions (e.g., 5′UTR, 3′UTR, upstream, downstream) may affect gene expression levels [34,35,36]. Because causal variants are expected to exhibit strong LD with the top SNP, variants showing both high *r*^2^ values and potentially functional annotations were considered strong candidates. Based on these criteria, 96 variants were selected for further validation (Table 2 and Table S1).

Among the 96 selected variants, 70 were successfully genotyped in 424 animals of the Hyogo population (*n* = 432) (Table S1). The remaining variants could not be genotyped mainly due to assay design failure or insufficient flanking sequence for primer design. Association analysis with FAR revealed that the most significant variant was a missense mutation in the *GGT1* gene (c.589G>A, p.Asp197Asn; *p* = 2.86 × 10^−6^; Table 3). The GGT1 gene encodes type I γ-glutamyltransferase, which plays a central role in glutathione metabolism [33]. Glutathione is a major antioxidant; however, one of its metabolic products, cysteinylglycine (Cys-Gly), has been reported to contribute to oxidative stress [34]. Oxidative stress may impair insulin signaling [37,38]. Under normal conditions, insulin suppresses lipolysis in adipocytes and limits the release of free fatty acids into circulation [39]. Impaired insulin signaling may therefore increase circulating free fatty acids and promote ectopic lipid deposition in skeletal muscle [40]. Accordingly, functional alteration of *GGT1* caused by the missense mutation may influence marbling formation through modulation of oxidative stress and insulin sensitivity. In addition, a 5′UTR variant in *GGT1* (c. −256G>T) showed a similarly low *p*-value (3.04 × 10^−6^; Table 3), suggesting a possible regulatory effect on gene expression. Both variants were in strong LD with the top SNP (*r*^2^ = 0.748), supporting their candidacy as potential causal variants underlying this QTL.

Gene function analysis also highlighted *SLC5A1* as another biologically plausible candidate within the candidate region (Table S3). *SLC5A1* encodes the sodium–glucose cotransporter 1 (SGLT1), which plays a key role in intestinal glucose absorption and has been implicated in the regulation of incretin secretion, including glucose-dependent insulinotropic polypeptide (GIP), from enteroendocrine cells [20,21]. While the relevance of this pathway to intramuscular fat deposition in cattle remains to be clarified, GIP signaling has been suggested to influence adipocyte differentiation and intramuscular adipose tissue development [41], providing a potential link between nutrient handling and marbling-related phenotypes. In addition, experimental work in mice has indicated that altered *SLC5A1* expression may affect signaling pathways related to growth and vascular responses, including the p70S6K/Akt/mTOR axis and VEGFA expression [42]. Because vascular remodeling and nutrient supply can be associated with adipose tissue expansion [43], such mechanisms could be relevant to the development of intramuscular adipose depots, although species- and tissue-specific differences should be considered. In our data, a missense variant in *SLC5A1* (c.32C>T, p.Thr11Met) showed a relatively strong association with FAR (*p* = 2.03 × 10^−5^; Table 3) and high LD with the top SNP (*r*^2^ = 0.775). Taken together, these observations nominate *SLC5A1* as a candidate gene at this locus; however, additional evidence—such as tissue-specific expression analyses and functional assays—will be needed to evaluate whether this variant contributes directly to variation in FAR.

## 4. Conclusions

The identified polymorphisms in GGT1 and SLC5A1 represent promising molecular markers for genetic selection aimed at optimizing marbling-related traits in Hyogo Japanese Black cattle. Although functional validation is still required to confirm the causal mutation and clarify the biological mechanisms involved, these findings contribute to a better understanding of the genetic architecture of fat deposition. Further validation in independent populations will help determine the broader applicability of this locus.

## Figures and Tables

**Table 1:** The five suggestive single-nucleotide polymorphisms (SNPs) in the quantitative trait locus (QTL) region on BTA17.

Position ^1^	SNP ID	SNP Name	*p*-Value
72,329,662	rs110240047	ARS-BFGL-NGS-28077	3.60 × 10^−6^
72,579,763	rs108976372	ARS-BFGL-NGS-17791	1.01 × 10^−5^
72,554,429	rs109808146	ARS-BFGL-NGS-62627	1.16 × 10^−5^
73,315,120	rs41856310	ARS-BFGL-NGS-117653	1.39 × 10^−5^
72,790,867	rs110840574	ARS-BFGL-NGS-105537	3.01 × 10^−5^

^1^ The positions were based on the bovine genome, UMD3.1.

**Table 2:** Number of polymorphisms selected for verification of effects on FAR.

Annotation	LD with Top SNP (*r*^2^)									Total Number of Polymorphisms	Total Number of Genes ^1^
	0.9–1.0	0.8–0.9	0.7–0.8	0.6–0.7	0.5–0.6	0.4–0.5	0.3–0.4	0.2–0.3	0.1–0.2		
stop_gained								1		1	1
missense_variant		1	3	3	4			4	5	20	14
5_prime_UTR_variant			3		2	1			1	6	3
3_prime_UTR_variant	2	1	2		2	1	1			9	7
upstream_gene_variant	2	3	5	6	8	2	3			29	17
downstream_gene_variant	2	1	6	2	5	1	3			20	15
splice_region_variant	1		2				2			5	4
synonymous_variant	2		1	2		1	1			7	5
Total	9	6	20	13	21	6	10	5	6	96	30

^1^ The total number of genes where the polymorphisms were located.

**Table 3:** Gene frequency and effects on FAR of candidate polymorphisms.

Polymorphism	Genotype Frequency			Allele Frequency		LD	*p*-Value	FAR (%) ^1^		
*GGT1* c.589G>A	AA	AG	GG	A	G			AA	AG	GG
	97	223	104	0.49	0.51	0.748	2.86 × 10^−6^	39.0 ^a^ ± 0.275	37.9 ^b^ ± 0.181	37.0 ^c^ ± 0.265
*GGT1* c. −256G>T	GG	GT	TT	G	T			GG	GT	TT
	92	228	104	0.49	0.51	0.748	3.04 × 10^−6^	39.1 ^a^ ± 0.279	37.8 ^b^ ± 0.180	37.1 ^b^ ± 0.264
*SLC5A1* c.32C>T	CC	CT	TT	C	T			CC	CT	TT
	145	215	64	0.60	0.40	0.775	1.67 × 10^−5^	38.8 ^a^ ± 0.225	37.5 ^b^ ± 0.186	37.2 ^b^ ± 0.334

^1^ FAR values are shown as the sum of the overall mean and the least square mean of residuals (e-values) for each genotype. Means with different superscripts (a–c) are significantly different between genotypes.

## Data Availability

The original contributions presented in this study are included in the article. Further inquiries can be directed to the corresponding author.

## Supplementary Materials

The following supporting information can be downloaded at: https://www.mdpi.com/article/10.3390/genes17040363/s1, Table S1: Primer and prove sequences for genotyping in BioMark system or KASP genotyping assay; Table S2: Number of polymorphisms located in the candidate region and showing LD (*r*^2^ > 0.1) with top SNP; Table S3: Function of genes located within candidate region and having polymorphisms of *r*^2^ > 0.1 with top SNP.
